# Generative artificial intelligence and terminology work: structural associations of prompt strategy preference, cognitive load, and self-efficacy

**DOI:** 10.3389/fpsyg.2026.1929666

**Published:** 2026-10-30

**Authors:** Kangxi Li, Liye Li, Jingwen Lu

**Affiliations:** School of Foreign Studies, Hefei University of Technology, Hefei, Anhui, China

**Keywords:** AI interaction self-efficacy, cognitive ergonomics, cognitive offloading, cross-sectional survey, human-AI collaboration, latent profile analysis, prompt strategy preference, subjective cognitive load

## Abstract

**Introduction:**

The widespread application of generative artificial intelligence (AI) in knowledge-intensive domains requires continuous adjustment of human cognitive patterns. In terminology work, aligning strict semantic standards against the non-deterministic output of large language models (LLMs) is closely intertwined with workers' subjective cognitive load. This study examines the structural associations among prompt strategy preference, AI interaction self-efficacy, and subjective cognitive load, while testing the potential moderating role of professional experience.

**Methods:**

Based on cross-sectional survey data from 1,760 practitioners using LLMs for terminology tasks, structural equation modeling (SEM) and latent profile analysis (LPA) were employed. SEM evaluated the linear structural associations among prompt strategy preference, self-efficacy, and cognitive load, while LPA identified behavioral user profiles. Multi-group equivalence testing was further conducted across three career stages (novices, intermediates, and experts).

**Results:**

Prompt strategy preference is significantly and negatively associated with subjective cognitive load, with AI interaction self-efficacy serving as a significant mediator in this structural relationship. LPA identified three distinct user profiles (low, medium, and high strategy use), with the high strategy group exhibiting the lowest cognitive load and highest self-efficacy. Multi-group equivalence tests indicated no statistically significant moderation across experience cohorts, demonstrating structural invariance of the core model across career stages.

**Discussion:**

From the perspective of cognitive ergonomics, upfront prompt structuring is structurally linked to downstream cognitive offloading and psychological buffering. These findings provide empirical evidence for individual cognitive resilience during human-AI collaboration in the generative AI era.

## 1 Introduction

The application of Large Language Models (LLMs) in knowledge-intensive domains not only changes the workflow of information generation but also imposes new requirements on the cognitive patterns of human workers (Dwivedi et al., 2023; Kasneci et al., 2023). In this digital transformation, terminology work emerges as a highly representative frontline scenario. Terminology work typically involves precise concept extraction, multilingual alignment, and domain knowledge definition, which highly depend on semantic precision and contextual constraints. As generative artificial intelligence intervenes in such professional tasks, practitioners have begun to delegate parts of retrieval and text validation to intelligent systems. However, the probabilistic and inherently non-deterministic output characteristics of LLMs create a distinct “cognitive tension” against the strict determinism required in this domain. This tension forces workers to continuously evaluate the algorithm's internal generative logic and repeatedly formulate prompts to correct the “knowledge hallucinations” it produces in the process of obtaining compliant terminology (Freire et al., 2023). Therefore, terminology work constitutes an ideal research context because its strict constraint requirements make workers' cognitive load highly sensitive to the quality of human-AI instruction interactions. Observing such human-AI collaborative scenarios reveals that transitioning from previous linear text queries to iterative dialogues with models improves initial generation efficiency while deriving new mental effort on the operator's end caused by uncertainty.

Currently, mainstream research in the fields of artificial intelligence and human-computer interaction mostly focuses on system performance evaluation, exploring which prompt frameworks can optimize machine-side output accuracy (Chen et al., 2025; Fatawi et al., 2024; Gao, 2023; Liu et al., 2023). However, systematic empirical evidence regarding the psychological states of human executors remains profoundly inadequate. First, methodologically, existing human-computer interaction studies predominantly rely on small-sample usability experiments or qualitative interviews, lacking large-scale quantitative evidence on users' cognitive states. Second, theoretically, current research over-relies on Technology Acceptance Models (TAM), focusing mainly on adoption intention while failing to deeply examine the cognitive processing costs borne by operators. Third, there is a variable selection bias toward objective efficiency and usability metrics, neglecting operators' psychological load and self-efficacy beliefs (Schulz et al., 2024). In human-machine systems, looking solely at the objective indicators of the tool side while ignoring the user's subjective stress makes it difficult to comprehensively measure the actual ergonomic efficacy of intelligent tools in modern workplaces. Faced with the unreliability of LLM response mechanisms, the changes in mental burden and the building of self-confidence when human users choose interaction strategies constitute research topics worthy of further investigation in the process of digital transformation.

To systematically address these research gaps, this study proposes three specific research questions: (1) Does prompt strategy preference exhibit a stable negative association with operators' subjective cognitive load? (2) Does AI interaction self-efficacy mediate this structural relationship? (3) Does the traditional professional dimension, years of experience, moderate these associations? In introducing these questions, prompt strategy preference is selected as the core independent variable because it represents the user's primary proactive method to constrain model uncertainty. Subjective cognitive load captures the fundamental mental cost of the human-AI tension, while self-efficacy is explicitly tested to uncover the psychological mechanism transforming operational strategy into cognitive relief. Distinct from research models that evaluate overall technology acceptance, this study aims to verify the co-variation direction of prompt strategy preference and subjective cognitive load through cross-sectional data observation. Specifically, this study explores the process of extracting latent user profiles through latent profile analysis (LPA) to understand the systematic differences in psychological load levels among groups with different strategy usage characteristics. In addition, this study examines whether the traditional dimension of professional background, “years of experience,” can play the expected buffering role against cognitive load within this structural mechanism, or whether it produces a skill-leveling effect.

Using a large-sample survey of 1,760 relevant practitioners, this study attempts to verify the effect of “cognitive offloading” in prompt strategies, provide quantitative evidence for establishing human-centered AI-assisted collaboration norms, and support the language services industry in optimizing employee ergonomic support strategies during the process of intelligent transformation.

## 2 Theoretical background and hypotheses development

### 2.1 Cognitive load transfer and structural mapping of prompt strategies

Cognitive load is one of the core indicators for evaluating task complexity and system usability. In the NASA-TLX evaluation system, cognitive load is usually composed of dimensions such as mental demand, temporal demand, effort, and frustration (Hart, 2006; Sweller, 1988). In terminology alignment and generation tasks, workers need not only to mobilize their own professional knowledge but also to plan prompt inputs to the LLM in working memory while continuously evaluating the consistency between model outputs and domain standards. This dual-processing process may constitute a high subjective cognitive load. For example, when extracting and translating a highly contextualized concept like “few-shot prompting” into a target language, a worker must not only retrieve linguistic equivalents but also instruct the LLM to adhere to specific domain glossary constraints. Without clear upfront instructions, the LLM might generate a literal but professionally inaccurate translation, forcing the user into a highly taxing cycle of subsequent manual corrections.

When using external information tools, individuals often externalize parts of memory, computation, or planning tasks to the tools to preserve limited cognitive resources (Hutchins, 1995; Paas et al., 2003). This mechanism is commonly referred to as cognitive offloading in cognitive science (Patac and Patac Jr, 2025). In LLM-based interactions, specific prompt strategies—such as persona prompting, few-shot prompting, chain-of-thought prompting, or setting clear constraints (Naser, 2025; Wei et al., 2022)—can be viewed as operational ways to guide the model to share reasoning and screening tasks. When workers tend to use structured prompt strategies, although they need to invest additional instruction design costs in the early stage of the task, these strategies may reduce the load mechanistically by imposing explicit boundary parameters on the algorithm. By defining specific contexts and formatting rules, structured prompts function as externalized logical scaffolds. This structural mapping process significantly minimizes the algorithmic search space and generative uncertainty, thereby sharply reducing the cognitive resources required for subsequent repeated corrections, ambiguity resolutions, and model hallucination checks throughout the entire task cycle. As recent empirical research indicates, compared to traditional unassisted trial-and-error, interactions driven by generative AI and precise instructions can effectively reduce the operator's cognitive load at the initial stage of multi-task divergence and provide significant creative psychological support (Noy and Zhang, 2023). Therefore, this study expects a negative association between prompt strategy preference and subjective cognitive load.

H1: The overall level of prompt strategy preference is significantly and negatively associated with the subjective cognitive load of terminology workers.

### 2.2 Latent user classification based on strategy habits

In addition to the overall correlation between variables, investigating the behavioral heterogeneity within the subject population helps to understand generative AI usage patterns in more detail. The actual application of technical tools is usually not uniformly distributed, and users often rely on different combinations of strategies (Dang et al., 2023). In terminology workflows, some subjects may use LLMs as extended search tools, employing only simple natural language instructions; other subjects may tend to adopt more complex interaction methods, such as multi-turn iteration, persona setting, few-shot examples, and constraint prompting (Zamfirescu-Pereira et al., 2023).

This difference in operating habits suggests that there may be several latent classes with different strategy usage patterns in the sample. Latent Profile Analysis (LPA) can identify groups with high internal homogeneity and obvious differences between classes based on the subject's score patterns on multiple continuous indicators. This study further hypothesizes that different latent profiles of prompt strategies may also show significant differences in average subjective cognitive load. Therefore, the following exploratory hypotheses are proposed:

H2a: When terminology workers interact with LLMs, their prompt strategy preferences can be identified as several latent profiles with significant differences.

H2b: There are significant differences in subjective cognitive load among different latent profiles of prompt strategies.

### 2.3 Transmission mechanism of AI interaction self-efficacy

On the basis of understanding the relationship between prompt strategies and cognitive load, self-efficacy provides an important perspective for explaining the user's internal psychological mechanisms. According to social cognitive theory (Bandura, 1977), an individual's judgment of whether they can organize and execute necessary actions to achieve goals in a specific context is an important factor affecting behavioral choices and emotional responses. In LLM workflows characterized by uncertain outputs, pure domain knowledge does not necessarily guarantee stable task performance. Users also need the ability to adjust prompts, interpret system feedback, and correct model outputs. This study defines “AI interaction self-efficacy” in this context as the user's confidence in their ability to effectively control, modify, and utilize AI outputs.

Individuals who tend to use high-level prompt strategies may develop a higher sense of system mastery through repeatedly obtaining effective feedback. The specific psychological mechanism through which self-efficacy mitigates subjective cognitive load relies on the proactive control over unreliability (Cai et al., 2022). When users efficiently utilize structured prompts, the resulting predictable outputs alleviate the uncertainty-related anxiety inherent in black-box AI tools. A high level of self-efficacy allows individuals to confidently interpret system feedback and rapidly distinguish between human instruction errors and inherent algorithmic limitations. Consequently, it improves decision-making efficiency, empowering them to strategically abandon ineffective trial-and-error attempts without experiencing severe psychological frustration. This cognitive clarity and reduced anxiety ultimately transmit into a significantly lower subjective cognitive load. Accordingly, the following hypotheses are proposed:

H3: Prompt strategy preference is significantly and positively associated with AI interaction self-efficacy.

H4: AI interaction self-efficacy plays a mediating role between prompt strategy preference and cognitive load.

### 2.4 Boundaries of professional experience constraints and the “Expert Interruption” phenomenon

Traditional cognitive load theory holds that long-term professional experience helps form stable domain schemata, thereby reducing individuals' cognitive load in complex tasks. In the context of using conventional computer-aided translation (CAT) tools, senior experts usually exhibit a cognitive load advantage in task processing due to their stronger schema recognition capabilities and domain knowledge reserves (Bowker and Ciro, 2019).

However, in generative AI scenarios, there may be boundaries to this rule. The output of LLMs does not completely follow the matching logic of traditional terminology databases or structured corpora; the generated results may be grammatically fluent but inconsistent with domain standards or conceptual frameworks. Therefore, when facing model hallucinations or semantic deviations, experts' original domain experience may not be directly translated into algorithmic interaction advantages (Lebovitz et al., 2022). This discrepancy hints at a potential “skill-leveling effect,” where the traditional cognitive advantages of expert schemata are disrupted by algorithmic opacity. If years of experience fail to significantly moderate the relationship between prompt strategies and cognitive load, it may suggest that the use of generative AI has, to some extent, weakened the role of traditional experience in buffering cognitive load. Based on this, the following hypothesis is proposed:

H5: Years of experience moderates the structural relationships in the context of prompt strategy usage, AI interaction self-efficacy, and subjective cognitive load, such that the mitigating effect of prompt strategies on cognitive load is stronger for senior workers than for novices.

Integrating the above theoretical background discussion and the development of various specific hypotheses into an organically integrated theoretical model, this study constructs a comprehensive theoretical conceptual model containing direct effects (grounded in Cognitive Load Theory), mediating mechanisms (explained by Social Cognitive Theory), and boundary moderating effects (framed by Cognitive Schema Theory) (as shown in Figure 1).

**Figure 1:**
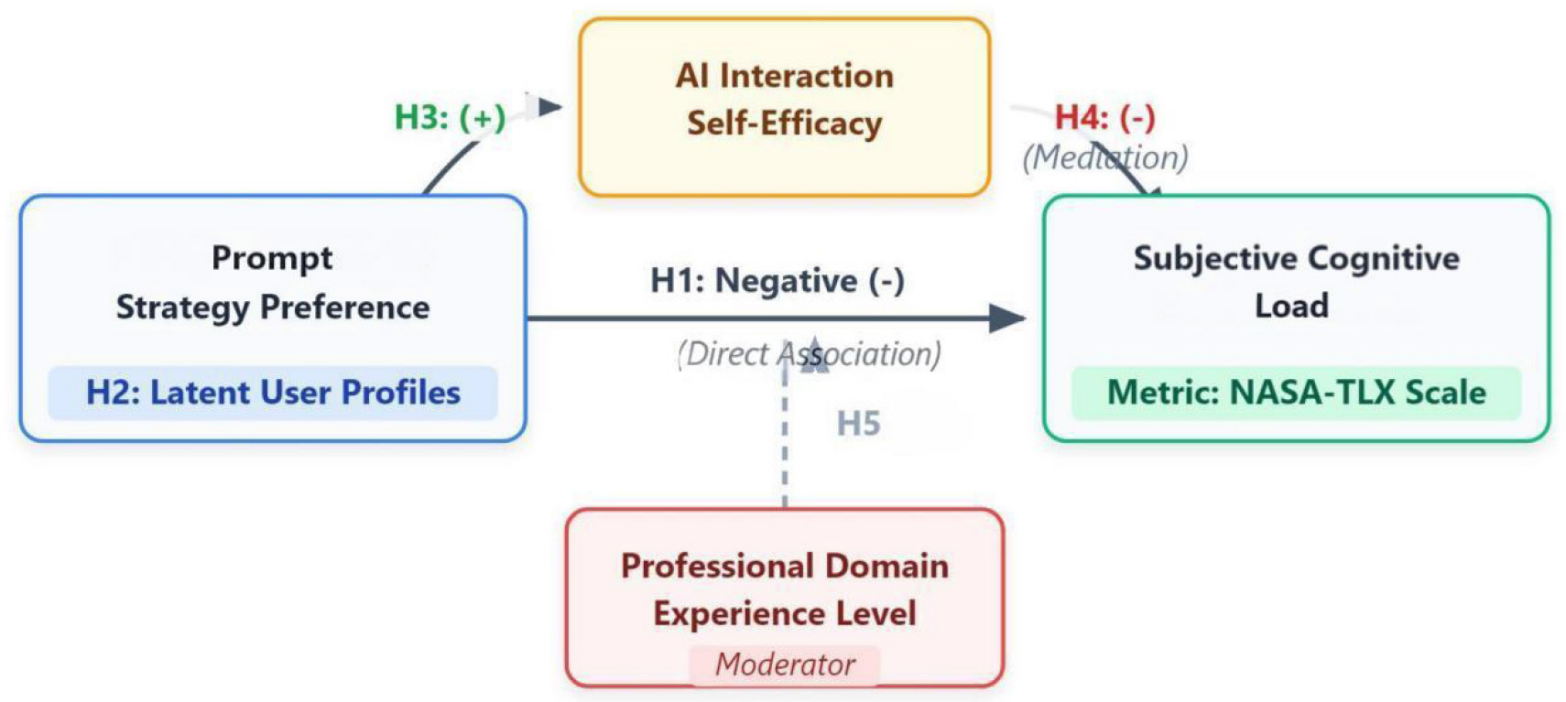
Theoretical framework and research hypothesis model of this study.

## 3 Methodology

### 3.1 Participants and procedure

This cross-sectional study aims to examine the prompt strategy usage characteristics and cognitive responses of terminology working groups in actual human-computer collaborative tasks. The target population was defined as professionals with practical experience in terminology extraction and professional translation, or with academic backgrounds in linguistics and computational linguistics. To ensure the alignment of the data with the research context, the inclusion criteria for the survey were restricted to: subjects must have actually used large language models, such as ChatGPT, Claude, etc., within the past 3 months to complete domain-specific terminology alignment, definition delineation, or related text validation tasks.

Data were distributed through an online survey platform and targeted professional language service networks (e.g., translation forums, terminology practitioner communities, and university alumni groups), during which a total of 1,920 initial questionnaires were retrieved. To reduce invalid responses and insufficient attention that self-administered questionnaires might bring, this study set up multiple data screening procedures during the implementation process. First, the system background automatically excluded samples with abnormal response durations (e.g., completion time of less than 120 s). Second, the researchers screened out straight-lining response patterns that showed a single fixed value on continuous Likert scales (defined as providing identical answers to 10 or more consecutive items). Finally, forced attention check questions (e.g., “Please select ‘Strongly Disagree' for this item to confirm your attention”) were embedded in the questionnaire, and those who failed to answer as required were excluded from the final analysis. After the above strict cleaning procedures, this study excluded 160 invalid questionnaires and finally obtained 1,760 valid samples, with a valid response rate of 91.67%. To ensure methodological transparency, the complete statistical syntax (including SPSS, AMOS paths, and Mplus code for LPA) has been comprehensive documented in Supplementary Material S2.

The demographic characteristics of the valid sample show a distinct pattern of knowledge-intensive industries. Female subjects accounted for 55.63%, and male subjects accounted for 41.76%. In terms of educational background, those with a master's degree or higher accounted for 95.23%. Regarding work experience, the novice group with less than 3 years of experience comprised 837 people (47.56%), the intermediate group with 3 to 5 years comprised 490 people (27.84%), and the expert group with 5 or more years comprised 433 people (24.60%). This distribution provides a relatively balanced sample base for the subsequent multi-group structural equation modeling test, as detailed in Table 1.

**Table 1:** Demographic characteristics of the participants.

Variables	Categories	Frequency (*N* = 1760)	Percentage (%)
Gender	Female	979	55.63
	Male	735	41.76
	Other/Prefer not to say	46	2.61
Education	Bachelor's or below	84	4.77
	Master's degree	1159	65.85
	Doctoral degree or higher	517	29.38
Occupation	Full-time terminologist/Terminology engineer	599	34.03
	Translator/Localization specialist	553	31.42
	Linguistics/Translation researcher	362	20.57
	Computational linguistics/NLP researcher	246	13.98
Work Experience	Novices (< 3 years of experience)	837	47.56
	Intermediates (3–5 years of experience)	490	27.84
	Experts (≥ 5 years of experience)	433	24.60
LLM Usage Frequency	1–2 times per week	569	32.33
	3–4 times per week	509	28.92
	About once a day/Multiple times a day	304	17.27
	Several times a month	378	21.48

### 3.2 Measures

All measurement tools used to evaluate latent variables were based on standardized questionnaires in the existing literature and were contextually revised to fit generative AI interaction scenarios. A 7-point Likert scale was uniformly adopted, with a value of 1 representing “strongly disagree” or “extremely low” and a value of 7 representing “strongly agree” or “extremely high”. The full items of all scales and demographic questions have been attached to the supplementary materials (see Supplementary Material S1).

#### 3.2.1 Prompt strategy preference scale (PESP-T)

This study developed the PESP-T scale based on the operational principles in algorithmic prompt optimization foundation corpus research by (Brown et al. 2020) and (Wei et al. 2022). Prior to the formal large-scale survey, the scale underwent a rigorous development procedure. The initial item pool was evaluated by a panel of three computational linguistics experts to ensure content validity. Subsequently, a pilot study (*N* = 120) was conducted. Exploratory Factor Analysis (EFA) on the pilot data confirmed that all seven strategy items loaded strongly onto a single dominant factor (explaining 65.4% of the total variance). Consequently, these seven strategies were identified as reflective indicators measuring an underlying unidimensional construct of strategy preference: persona setting (P1), few-shot guidance (P2), chain of thought (P3), corpus-driven (P4), iterative feedback (P5), constraint instructions (P6), and structured instructions (P7). For example, for dimension P3, the item is phrased as: “I will ask the model to show its reasoning steps before giving an answer”; dimension P6 is defined as: “I will clearly list prohibited items or boundary conditions to follow specific domain standards.”

#### 3.2.2 Cognitive load scale (NASA-TLX)

Subjective cognitive load was measured using the Raw NASA-TLX scale revised by (Hart 2006). The scale comprises six single-item dimensions designed to capture different aspects of the operator's subjective experience: mental demand (N1), temporal demand (N2), effort (N3), frustration level (N4), physical demand (N5), and self-reported performance (N6). In this study, the task descriptions within the items were systematically contextualized to reflect the LLM-assisted terminology processing workflow. For instance, the mental demand (N1) item was framed as: “How much mental and cognitive activity was required to formulate prompts, comprehend AI outputs, and make professional judgements throughout the interaction?” Since a high score in self-reported performance (N6) originally represented high satisfaction (which is opposite to the direction of distress/load), this item was reverse-scored (computed as 8–original score) prior to statistics, ensuring that higher scores across all items uniformly indicate greater subjective cognitive load. Following the established protocol for the unweighted Raw-TLX (Hart, 2006), the overall subjective cognitive load was derived by calculating the arithmetic mean of all six items. While the self-reported performance dimension captures subjective task outcomes rather than pure mental exertion, its reverse-scored inclusion is maintained in this study. This unified aggregation aligns with standard human-computer interaction practices, as the user's perception of poor performance and cumulative physical fatigue often directly exacerbates the overall psychological strain experienced during unpredictable AI interactions. Furthermore, to ensure the robustness of the cognitive load measurement, a sensitivity analysis was conducted by excluding the physical demand (N5) and self-reported performance (N6) items. The structural model results utilizing this adjusted four-item NASA-TLX construct remained consistent with the primary findings (the detailed sensitivity analysis outputs are provided in Supplementary Material S2).

#### 3.2.3 Covariate measure matrix

To control for relevant external factors and test the mediating mechanisms, this study introduced several auxiliary measures. AI interaction self-efficacy (SE1–SE5) was adapted from Compeau and Higgins' (1995) Computer Self-Efficacy Scale, with a representative item being: “I am confident in correcting model outputs by redesigning instructions.” Terminology competence (C1–C5) was adapted from studies by (Beeby et al. 2003) and (Arntz et al. 2016) to measure subjects' mastery of domain concept networks. Task complexity (T1–T4) was revised based on Wood's (1986) task complexity framework. System trust (TR1–TR5) was adapted from the Human-Computer Trust Scale by (Jian et al. 2000), where items regarding concerns about algorithmic hallucinations were reverse-scored according to the scale's direction. Perceived output quality (Q1–Q5) was revised based on DeLone and McLean's (2003) Information Systems Success Model.

### 3.3 Data analytical strategy

This study adopts a multi-stage statistical modeling scheme. First, descriptive statistics, data cleaning, and two-tailed Pearson correlation analyses were performed using SPSS 26.0. Second, to identify the cohort heterogeneity in prompt strategy usage patterns, Latent Profile Analysis (LPA) was conducted on the scores of each PESP-T item, and the optimal number of classes was determined based on information criteria, entropy, class interpretability, and model parsimony. Subsequently, one-way analysis of variance (ANOVA) was used to compare the mean differences in cognitive load and AI interaction self-efficacy among different latent classes.

Methodologically, structural equation modeling (SEM) and LPA were employed as two orthogonal but highly complementary analytical tracks to provide a comprehensive understanding of the core construct. While SEM treats prompt strategy preference as a continuous latent variable to evaluate the degree of its linear structural associations with cognitive load and self-efficacy (a variable-centered approach), LPA treats the strategy items as manifest variables to identify distinct behavioral types within the population (a person-centered approach). Combining these two theoretically compatible treatments effectively allows this study to simultaneously map the overarching transmission mechanism and uncover the operational heterogeneity of different user cohorts.

In the structural relationship testing phase, AMOS was used to construct a structural equation model (SEM), and model parameters were estimated using the maximum likelihood method, with relevant covariates systematically controlled to ensure the robustness of the main paths. To test the mediating effect, this study used 5,000 Bootstrap resamples to calculate the indirect effect and its confidence intervals (Hayes, 2017). Finally, using work experience as the grouping variable, a Multi-Group Structural Equation Model (Multi-Group SEM) was constructed to test whether there were differences in core paths between experience groups by comparing the fit differences between the unconstrained model and the path-constrained model.

## 4 Results

### 4.1 Reliability, validity assessment of measurement tools, and common method bias test

Before hypothesis testing, this study first tested the reliability and validity of the core measurement tools. Preliminary sample suitability tests showed that the Kaiser-Meyer-Olkin (KMO) value of the total sample was 0.907, and Bartlett's test of sphericity reached an extremely significant level (χ^2^ = 25591.08, *df* = 666, *p* < 0.001), indicating that the sample data were highly suitable for latent variable factor extraction. In terms of multicollinearity screening, the variance inflation factor (VIF) among independent variables was at 1.241 (for Perceived Output Quality), which is far below the conventional test threshold of 5.0 (Hair et al., 2019).

The measurement model of Confirmatory Factor Analysis (CFA) showed good structural characteristics. Specifically, all standardized factor loadings for the measurement items ranged from 0.67 to 0.77 (all significant at *p* < 0.001), indicating strong indicator reliability. Combining the detailed test reports in Table 2, it can be seen that the Cronbach's α and composite reliability (CR) of each core scale were far higher than the conventional standard of 0.70, showing excellent internal consistency; meanwhile, the average variance extracted (AVE) of most latent variables reached or exceeded the threshold of 0.50. Although the AVE for Task Complexity (T, AVE = 0.432) and Subjective Cognitive Load (N, AVE = 0.476) fell slightly below the 0.50 threshold, their Composite Reliability (CR) values (0.753 and 0.845) were well above 0.70, which justifies acceptable convergent validity according to (Fornell and Larcker 1981). Regarding discriminant validity, Table 3 and Figure 2 demonstrates that the square root of each construct's AVE was larger than any of its longitudinal or transverse correlations, satisfying the (Fornell and Larcker 1981) criterion. Furthermore, to provide a more rigorous assessment, the Heterotrait-Monotrait Ratio (HTMT) values across all latent variable pairs were evaluated. The maximum HTMT value obtained was strictly below the conservative threshold of 0.85 (Henseler et al., 2015), providing robust confirmation of discriminant validity.

**Table 2:** Convergent validity and internal reliability reports of each dimension.

Latent variable (construct)	Indicators covered	Cronbach's α	Composite reliability (CR)	Average variance extracted (AVE)
Prompt strategy preference (P)	7	0.891	0.891	0.538
Subjective cognitive load (N)	6	0.845	0.845	0.476
AI interaction self-efficacy (SE)	5	0.851	0.851	0.533
Terminology competence (C)	5	0.857	0.857	0.545
Perceived output quality (Q)	5	0.862	0.862	0.556
System trust concerns (TR)	5	0.835	0.835	0.503
Task complexity (T)	4	0.753	0.753	0.432

**Table 3:** Discriminant validity test: Fornell–Larcker matrix.

Variables	P	N	SE	C	Q	T	TR
P: Prompt strategy preference	**0.734**						
N: Subjective cognitive load	−0.156^***^	**0.690**					
SE: AI interaction self-efficacy	0.273^***^	−0.242^***^	**0.730**				
C: Terminology competence	0.167^***^	−0.084^***^	0.237^***^	**0.738**			
Q: Perceived output quality	0.328^***^	−0.258^***^	0.366^***^	0.134^***^	**0.745**		
T: Task complexity	−0.003	0.244^***^	0.001	−0.018	−0.054^*^	**0.657**	
TR: System trust concerns	0.140^***^	−0.005	0.046	0.007	0.020	0.050^*^	**0.709**

Bold values on the diagonal are the square root of the AVE of each latent variable; off-diagonal values are the Pearson correlation coefficients between latent variables. ^*^p < 0.05, ^***^p < 0.001.

**Figure 2:**
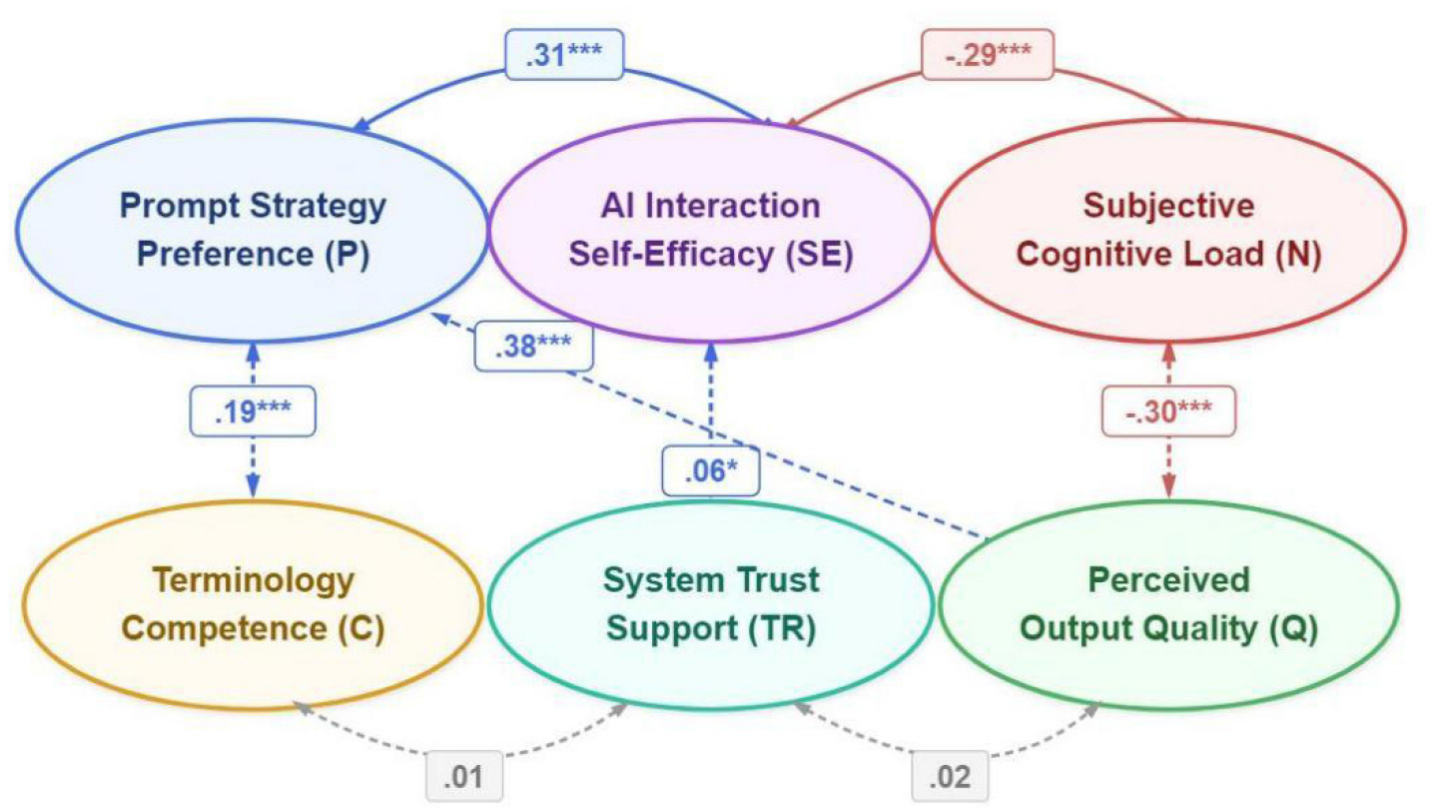
Structural correlation and path summary diagram of the latent construct model.

Since the data in this study came from self-report questionnaires at a single point in time, common method bias may exist. Harman's single-factor test showed that the variance explained by the first factor in the unrotated principal component analysis was 17.96%, which is lower than the common threshold of 50%. Furthermore, to provide a more robust structural assessment beyond Harman's test, this study conducted an Unmeasured Latent Method Construct (ULMC) sensitivity analysis by introducing a common method factor into the CFA model. The comparison indicated that adding this method factor did not yield a significant improvement in model fit (Baseline model: χ^2^ = 972.8, *df* = 608; ULMC model: χ^2^ = 959.0, *df* = 607; ΔCFI = 0.003, ΔRMSEA = 0.002, both strictly below the 0.01 threshold). Meanwhile, all substantive trait factor loadings remained highly significant with negligible changes in their magnitudes (Δλ < 0.05). Therefore, common method bias does not pose a serious threat (Podsakoff et al., 2003).

### 4.2 Classification of prompt strategy use patterns based on latent profile analysis

To examine the group heterogeneity of terminology workers in prompt strategy usage, this study conducted a Latent Profile Analysis (LPA) on the 7 items of the prompt strategy preference scale. After comparing the 2-class, 3-class, and 4-class models sequentially, the results showed that as the number of classes increased, both AIC and aBIC decreased, with the 4-class model having the lowest AIC and aBIC. However, the 3-class model had the highest entropy (Entropy = 0.865). The likelihood ratio test results showed that the fit of the 3-class model was significantly improved compared to the 2-class model (LMR-LRT *p* = 0.012; BLRT *p* < 0.001), while the 4-class model did not reach statistical significance in LMR-LRT or BLRT compared to the 3-class model (*p* > 0.05). Considering information criteria, entropy, classification clarity, and model parsimony comprehensively (Nylund et al., 2007), this study finally selected the 3-class model as the follow-up analysis scheme. The detailed model comparison results are shown in Table 4.

**Table 4:** Comparison results of 2- to 4-class latent profile models.

**Number of classes**	AIC	aBIC	Entropy	LMR-LRT (*p*)	BLRT (*p*)
2-Class model	32152.12	32185.34	0.781	< 0.001	< 0.001
3-Class model	28912.42	28975.21	0.865	0.012	< 0.001
4-Class model	28842.15	28892.44	0.812	0.245 (ns)	0.180 (ns)

LMR-LRT, Lo-Mendell-Rubin Likelihood Ratio Test; BLRT, bootstrapped likelihood ratio test; ns, non-significant difference between groups (*p* > 0.05).

Although the 4-class model had the lowest AIC and aBIC, the 3-class model had higher entropy, clearer classification distinction, and better theoretical interpretability. Therefore, this study chose the 3-class model as the final classification scheme.

According to the mean score patterns of the three classes of subjects on the 7 prompt strategy indicators, this study named them the low strategy use group, medium strategy use group, and high strategy use group, respectively. The low strategy use group contained 470 people (26.7%); the medium strategy use group contained 875 people (49.7%); and the high strategy use group contained 415 people (23.6%). From the class characteristics, the low strategy use group rarely used structured strategies such as persona setting, example guidance, chain-of-thought prompting, and constraint instructions; the medium strategy use group was close to the midpoint of the scale on most indicators; and the high strategy use group exhibited high usage levels on all prompt strategy indicators. The score patterns of the three groups on each prompt strategy item are shown in Figure 3.

**Figure 3:**
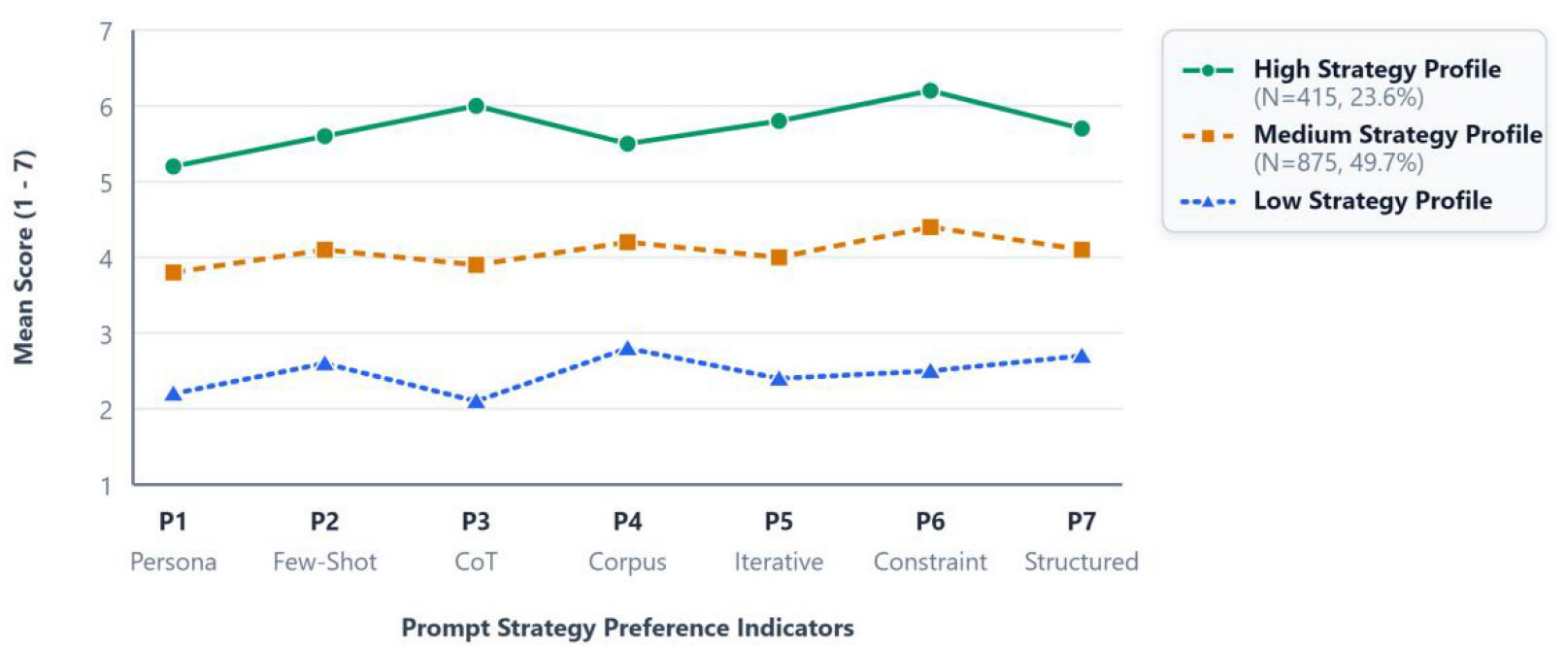
Mean distribution of three types of prompt strategy users on 7 prompt strategy indicators.

One-way ANOVA was further used to compare the differences in subjective cognitive load and AI interaction self-efficacy among the three groups. To display the mean differences and data distribution density of psychological characteristics among people with different strategy preferences more comprehensively, this study plotted a Raincloud Plot (as shown in Figure 4). The analysis results confirmed that there were significant differences among the three groups in subjective cognitive load (*F*(2, 1757) = 19.07, *p* < 0.001) and AI interaction self-efficacy (*F*(2, 1757) = 52.67, *p* < 0.001). As the distribution diagram visually shows, the low strategy use group bore the highest cognitive load and had the lowest sense of system control efficacy; conversely, the high strategy use group not only had a significantly lower center of gravity in cognitive load distribution but also exhibited the highest overall level of AI interaction self-efficacy.

**Figure 4:**
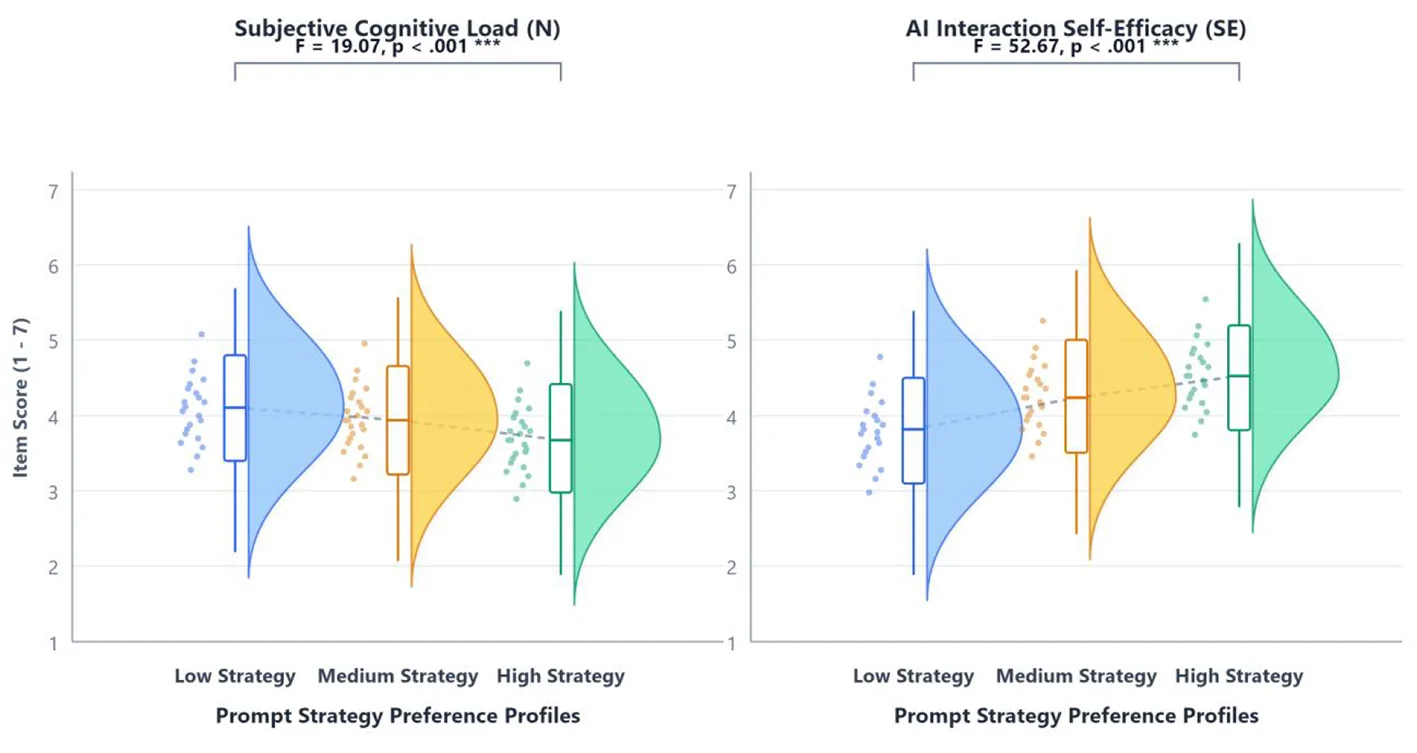
Difference distribution of three latent profiles of prompt strategies in subjective cognitive load and AI interaction self-efficacy (Raincloud Plot).

Overall, the LPA results support H2a, there are distinguishable latent profiles of terminology workers in prompt strategy usage; the ANOVA results further support H2b, there are significant differences in subjective cognitive load among different prompt strategy usage types. Meanwhile, the differences in self-efficacy between classes also provide a preliminary basis for subsequent mediation model analysis.

### 4.3 Descriptive statistics and correlation matrix of major variables

Before testing the structural equation model, descriptive statistics and Pearson correlation analysis were first performed on the major variables, as shown in Table 5. The mean of prompt strategy preference was *M* = 4.10, *SD* = 1.01; the mean of subjective cognitive load was *M* = 3.91, *SD* = 1.06; and the mean of AI interaction self-efficacy was *M* = 4.19, *SD* = 1.08.

**Table 5:** Descriptive statistics and pearson correlation matrix of major variables.

Variables	M	SD	1	2	3	4	5	6	7
1. Prompt strategy preference P	4.10	1.01	1						
2. Subjective cognitive load N	3.91	1.06	−0.156^***^	1					
3. AI interaction self-efficacy SE	4.19	1.08	0.273^***^	−0.242^***^	1				
4. Terminology competence C	4.00	1.05	0.167^***^	−0.084^***^	0.237^***^	1			
5. Perceived output quality Q	4.00	1.11	0.328^***^	−0.258^***^	0.366^***^	0.134^***^	1		
6. Task complexity T	4.20	1.07	−0.003	0.244^***^	0.000	−0.018	−0.054^*^	1	
7. System trust TR	3.79	1.08	0.140^***^	−0.004	0.046	0.007	0.020	0.050^*^	1

*M*, Mean; *SD*, Standard Deviation. Off-diagonal values in the table are Pearson correlation coefficients. ^*^ *p* < 0.05,^***^p < 0.001.

Correlation analysis results showed that prompt strategy preference was significantly and negatively correlated with subjective cognitive load, *r* = −0.156, *p* < 0.001, and significantly and positively correlated with AI interaction self-efficacy, *r* = 0.273, *p* < 0.001. AI interaction self-efficacy was also significantly and negatively correlated with subjective cognitive load, *r* = −0.242, *p* < 0.001. Task complexity (T) was significantly and positively correlated with subjective cognitive load (*r* = 0.244, *p* < 0.001), suggesting that more complex terminology tasks are associated with greater cognitive strain. These results are highly consistent with the theoretical expectations of this study and provide a preliminary basis for the subsequent mediation model test.

### 4.4 Overall structural equation model and mediation effect test

To test the structural relationships between the core latent variables and the mediating role of AI interaction self-efficacy, this study constructed a structural equation model (SEM) using the Maximum Likelihood estimation method. To ensure the rigor of the estimations in addressing potential confounding effects, both unadjusted and fully adjusted models were concurrently evaluated. In the adjusted model, all relevant demographic and task-contextual covariates (specifically controlling for age, education, task complexity, and system trust concerns) were systematically retained and evaluated to ensure the robustness of the main paths. The results indicated that the inclusion of covariates significantly contributed to the overall explanatory power, accounting for substantial variance in the endogenous variables (e.g., *R*^2^ = 0.084 for AI Interaction Self-Efficacy, and *R*^2^ = 0.132 for Subjective Cognitive Load). More importantly, the direction and significance of the core structural parameters and total effects remained exceptionally stable between the unadjusted and adjusted models. To determine the optimal theoretical framework, this study compared Hypothesized Model A (including both the direct path from prompt strategy preference to cognitive load and the mediating path through self-efficacy) with Competing Model B (constraining the direct path from prompt strategy preference to cognitive load to zero; i.e., a full mediation model) (the goodness-of-fit comparison is shown in Table 6). The results showed that all fit indices of Hypothesized Model A reached an excellent fit to the data when evaluated against conventional strict thresholds (e.g., χ^2^/*df* < 3.0, CFI ≥ 0.95, TLI ≥ 0.95, and RMSEA < 0.05), and the overall fit was significantly better than Competing Model B. Therefore, this study accepted Model A as the final explanatory model, and all subsequent parameters are reported based on the fully adjusted Model A.

**Table 6:** Comparison of structural equation model goodness-of-fit.

Model classification	χ^2^/*df*	CFI	TLI	RMSEA	SRMR
Hypothesized model A (including mediation effect)	1.60	0.985	0.984	0.018	0.032
Competing model B (excluding competitive path)	1.62	0.985	0.984	0.019	0.037

The standardized path coefficients and hypothesis testing results of the final Model A are detailed in Table 7. The analysis confirmed that prompt strategy preference has a significant positive predictive effect on AI interaction self-efficacy (supporting H3); while AI interaction self-efficacy significantly and negatively predicts subjective cognitive load. After controlling for the mediating effect of self-efficacy, the direct path coefficient from prompt strategy preference to subjective cognitive load remains significantly negative (supporting H1).

**Table 7:** Standardized path coefficients and mediation effect test of structural equation model.

Structural path category	Unadjusted model (β)	Fully adjusted model A (β)^*^	*p*-value	Bootstrap 95% CI	Hypothesis verification
H3: Prompt strategy preference P → AI interaction self-efficacy SE	0.312	0.290	< 0.001	[0.242, 0.335]	Supported
AI Interaction self-efficacy SE → Subjective cognitive load N	−0.224	−0.211	< 0.001	[-0.259, −0.165]	Significant
H1 (Direct effect): prompt strategy preference P → Subjective cognitive load N	−0.115	−0.101	< 0.001	[-0.150, −0.055]	Supported
H4 (Indirect effect): P → SE → N	−0.070	−0.061	< 0.001	[−0.078, −0.046]	Supported
Total effect: P → Cognitive load N (Direct + Indirect)	−0.185	−0.162	< 0.001	[−0.208, −0.113]	Significant

The adjusted model systematically controlled for demographic and task complexity covariates. Standardized estimates for Unadjusted Model are provided for sensitivity comparison.

For the mediating effect (H4), this study used the bias-corrected non-parametric Bootstrap method (5,000 resamples) to calculate the indirect effect. The data showed that the indirect effect of prompt strategy preference on reducing subjective cognitive load through AI interaction self-efficacy was significantly negative, and its 95% confidence interval strictly excluded 0 (95% CI: [−0.078, −0.046]). This indicates that AI interaction self-efficacy plays a significant partial mediating role between the two, verifying H4.

To present the above path relationships more intuitively this study further graphically represented the numerical results of the model (as shown in Figure 5). In the structural path model, the core independent variable “prompt strategy preference (P)” directly mitigates “subjective cognitive load (N)” (β = −0.101, *p* < 0.001), while simultaneously exerting an indirect effect mediated by “AI interaction self-efficacy (SE)” (β = −0.061, *p* < 0.001). This result indicates that the core “strategy-efficacy-load” transmission mechanism constructed in this study still has solid robustness and explanatory power after excluding the interference of external multi-source confounding variables.

**Figure 5:**
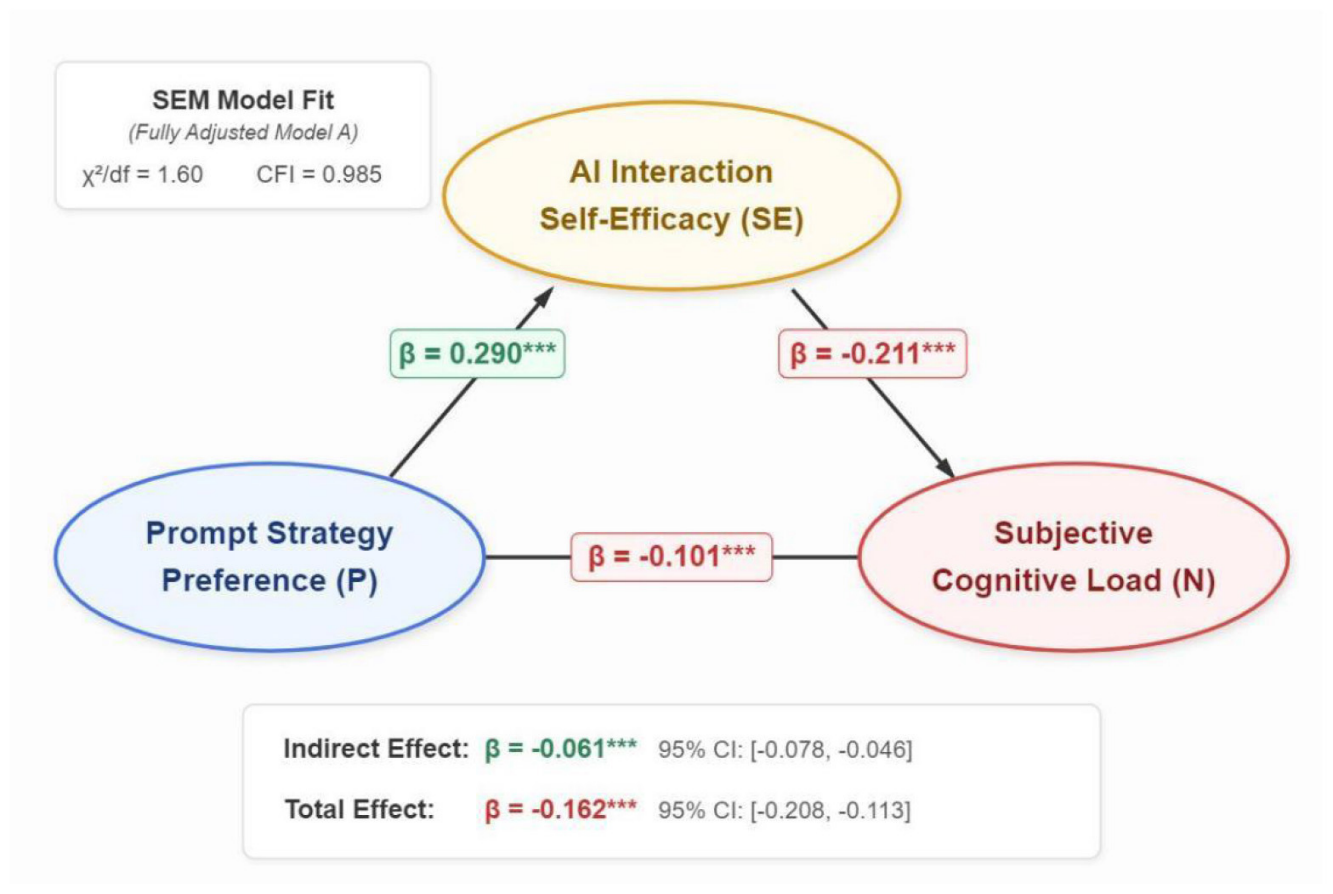
Standardized path coefficients and test results of the structural equation model for the full sample.

### 4.5 Multi-group analysis of background differences in professional experience

To explore whether professional experience acts as a boundary constraint within this conceptual framework (testing H5), the sample was classified into three distinct stages based on years of terminology-related work or study: Novices (less than 3 years, *N* = 837), Intermediates (3 to 5 years, *N* = 490), and Experts (5 years and above, *N* = 433, by merging the 5–10 years and more than 10 years cohorts). A Multi-Group Path Analysis was subsequently constructed across these three career stages to examine the equivalence of the structural path coefficients. Prior to evaluating these path differences, a multi-group CFA was routinely performed to confirm measurement invariance (MI) across the three cohorts. The sequential tests confirmed strong invariance, as the incremental changes in model fit indices safely remained within the conventional thresholds (e.g., ΔCFI < 0.01, ΔRMSEA < 0.015). Detailed configural, metric, and scalar model-fit results are reported in Section 8 of Supplementary Material S2. With the conceptual equivalence firmly established across novices, intermediates, and experts, the structural path comparisons were reliably interpreted.

The standardized path estimates and group comparison results are presented in Table 8. As indicated, the structural relationship patterns revealed varying within-group significance levels in the direct pathway from prompt strategy preference to cognitive load. First, the positive relationship between prompt strategy preference and AI self-efficacy was robust and highly significant for Novices (β = 0.279, *p* < 0.001), Intermediates (β = 0.297, *p* < 0.001), and Experts (β = 0.306, *p* < 0.001). Second, self-efficacy significantly mitigated cognitive load across all three groups. However, the direct association of prompt strategy preference on cognitive load was only statistically significant for Novices (β = −0.090, *p* = 0.012) and Intermediates (β = −0.130, *p* = 0.007), whereas it became non-significant for Experts (β = −0.080, *p* = 0.156).

**Table 8:** Multi-group path comparison across career developmental stages.

Structural association path	Novices (*N* = 837)	Intermediates (*N* = 490)	Experts (*N* = 433)	Path Difference Significance (pdiff)	Hypothesis evaluation
P: Prompt strategy → SE: AI Self-efficacy	0.279^***^	0.297^***^	0.306^***^	*p* > 0.05 (ns)	Supported / equivalent
P: Prompt strategy → N: Cognitive load	−0.090^*^	−0.130^**^	−0.080 (ns)	*p* > 0.05 (ns)	Not supported
SE: AI Self-efficacy → N: Cognitive load	−0.222^***^	−0.225^***^	−0.176^***^	*p* > 0.05 (ns)	Supported / equivalent
Control covariates	Excluded	Excluded	Excluded	–	–

β represents standardized path coefficients; ns, not significant. pdiff indicates the statistical significance of the Wald χ^2^difference test comparing the corresponding path coefficients across the three cohorts. Note that while within-group significance levels may visually vary, the Wald constraint tests confirm that the absolute numerical differences between these path coefficients across the three groups are not statistically significant. ^*^p < 0.05, ^**^p < 0.01, ^***^p < 0.001.

Most importantly, in conducting the cross-group parameter constraint tests an overall equivalence test was first performed by simultaneously constraining all core structural paths to be equal across the three experience cohorts. The Wald χ^2^ differences were evaluated against the unconstrained baseline model. This global test yielded no statistically significant path discrepancies among novices, intermediates, and experts for the model's core paths (*p* > 0.05). The multi-group equivalence test demonstrates that the fundamental structure of the model is statistically equivalent across different career stages. Therefore, H5—which predicted that the mitigating effect of prompt strategy on cognitive load would be stronger among senior experts—was not supported. Although within-group observations show that the direct cognitive offloading association of prompt strategy reached statistical significance for novices and intermediates but fell short of the significance threshold for experts, constrained structural tests confirm that the numerical differences between these paths do not meet the statistical criteria for divergence. This means it cannot be inferred that the underlying mechanism undergoes a fundamental qualitative or developmental transition across stages.

To present this symmetry in the interaction paths across the career span more intuitively, a comparative visualization of the standardized structural paths was evaluated (as shown in Figure 6). Although the direct cognitive offloading path displays varying significance levels within individual cohorts (as tentatively indicated by the asterisks), the formal equivalence testing confirms that the overall structure does not fundamentally diverge. Consequently, rather than a fragmented, stage-specific phenomenon, the overarching analysis underscores a broadly stable and universal psychological mechanism shared by terminology workers across different stages of their careers.

**Figure 6:**
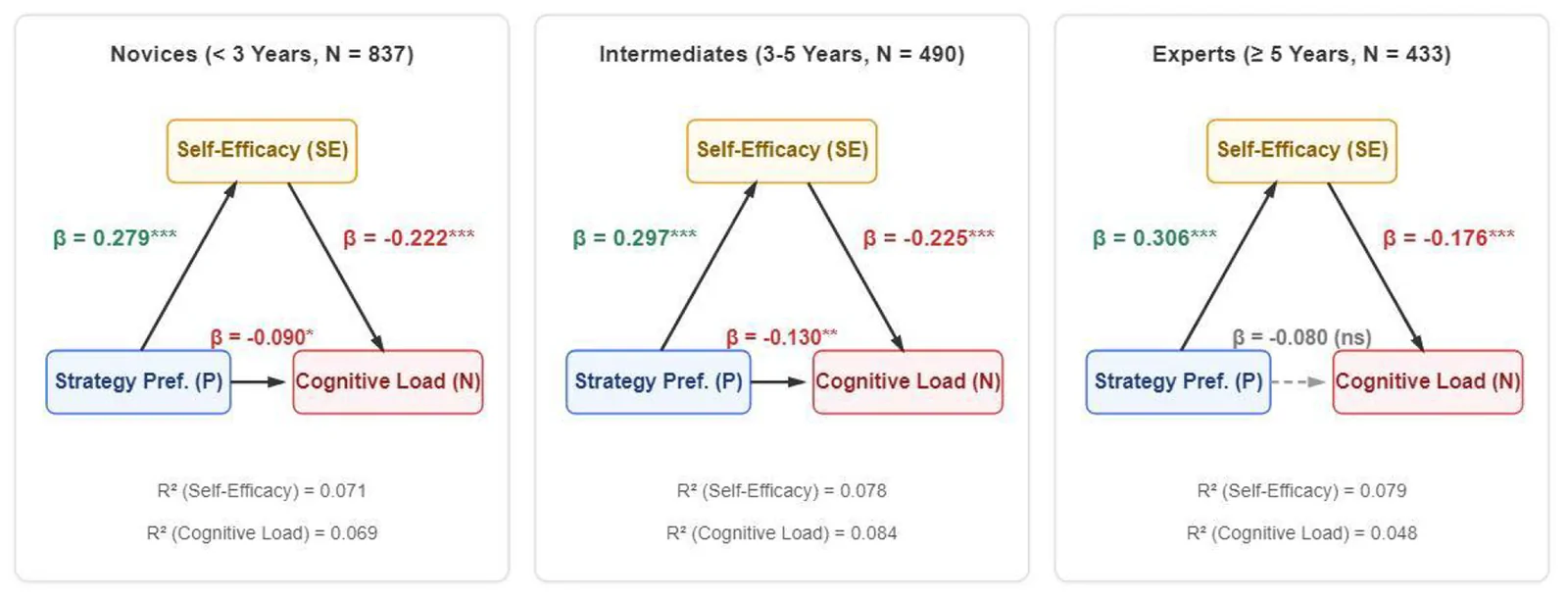
Multi-group comparison of structural path coefficients across career stages.

## 5 Discussion

Based on cross-sectional data from 1,760 terminology workers, this study used structural equation modeling (SEM) and latent profile analysis (LPA) to examine the multidimensional structural relationship among prompt strategy preference, AI interaction self-efficacy, and subjective cognitive load in the context of generative large language model assistance. The data analysis results supported most of the theoretical hypotheses of this study and suggested that the role of traditional professional experience in mitigating cognitive load may be weakened when exploring experience boundaries. The results of this study provide quantitative empirical evidence for understanding the cognitive coping mechanisms of knowledge workers in the era of generative artificial intelligence.

### 5.1 Main effect: quantitative support for the cognitive offloading mechanism

The path coefficients of the structural equation model indicate that structured and deep prompt strategy preferences are significantly and negatively associated with the subjective cognitive load level of workers. In actual terminology extraction and concept alignment tasks, the core challenge faced by practitioners is usually semantic ambiguity control in specific contexts. When users prefer to introduce explicit rules such as Chain-of-Thought or Few-shot in prompts, while requiring upfront working memory resources for instruction design, this strategic tendency ultimately exhibits a strong inverse association with the overall task-related cognitive load.

This phenomenon reveals a mechanism of cognitive resource allocation during the interaction process: well-structured upfront instruction design acts to constrain the generation space of large language models (LLMs), thereby substantially lowering the reported burden of subsequent evaluation and error-checking phases caused by algorithmic hallucinations. Although the cumulative retrospective measurement adopted in this study objectively reflects the “net load” after the accounting for the psychological requirements in the two stages, the negative result of this cumulative effect strongly confirms that the cognitive resources saved in the later verification stage far exceed the extra effort in the upfront writing stage in terms of total volume.

This negative association theoretically fits the “cognitive offloading” mechanism. In open-ended interactions lacking clear constraints, users must rely heavily on their working memory to compare machine output with professional standards in real time after each interaction. In contrast, pre-setting specific rules and boundary conditions in prompts essentially makes part of the implicit rule verification task explicit and “outsources” it to the algorithm's computational space as known parameters. Meanwhile, this study found that AI interaction self-efficacy plays a significant partial mediating role in the above structure. This means that the association between mitigation of cognitive load and prompt strategies is not only reflected at the physical operation level (such as reducing correction rounds) but also includes a psychological transmission mechanism: individuals who tend to use high-level strategies often possess a higher sense of system control; this belief in efficacy can act as an internal psychological resource that buffers the anxiety or frustration caused by model uncertainty, thus statistically corresponding to a lower overall cognitive load. This main effect perspective highlights another pertinent psychological attribute of prompt strategy optimization.

### 5.2 Latent profile differences: analysis of operational heterogeneity

Compared to examining the linear relationship of the overall sample, Latent Profile Analysis (LPA) reveals the heterogeneous distribution of strategy preferences among different types of users. The three classes of users extracted exhibited significant mean differences between groups: the “high strategy use group,” which accounted for about a quarter of the sample, scored the lowest on subjective cognitive load indicators and the highest on AI interaction self-efficacy.

In response to this group differentiation, analysis can be carried out in combination with specific terminology work contexts. The “low strategy use group,” which remains at the primary instruction interaction level, often directly relies on the default response logic of the LLM. This shallow interaction mode allows the algorithm's “black box” to diverge freely (Khosravi et al., 2022), resulting in highly unstable output quality, which in turn generates high cognitive costs in the later evaluation and error-checking stages. In contrast, the “high strategy use group” tends to implement strict formatting rules and persona framing at the starting stage. The process of using complex strategies essentially reflects the operator's behavioral tendency to implement predictive control over non-deterministic systems. By setting strict system boundary conditions, users not only converge the output framework of the model but also strengthen their sense of control over the task environment at the psychological level. The analysis of variance between groups confirms that this set of strategic behaviors aimed at enhancing environmental certainty corresponds to more positive psychological feedback levels and lower cognitive pressure on the user's part.

### 5.3 Boundary testing of professional experience: structural invariance and exploratory trends in cognitive offloading

The multi-group analysis of industry seniority involves a classic proposition in cognitive science and traditional occupational training. Traditional cognitive load theory usually holds that long-term domain schema accumulation gives senior experts a significant resource advantage when handling complex tasks, meaning they should exhibit lower mental depletion evaluations under equivalent task demands. However, in the cross-group equivalence test of this study, novices, intermediates, and seasoned experts did not show statistical differences in the overall core transmission paths of “strategy-self-efficacy-cognitive load.” This implies a broad structural invariance across different experience levels. While the overarching transmission framework remains statistically equivalent across seniority cohorts, observing the numerical coefficients within each group presents a subtle, exploratory trend regarding the direct pathway between prompt strategy and cognitive load.

From a descriptive standpoint, this numerical trend can be interpreted through the lens of cognitive schema theory and the evolving role of external scaffolding. For practitioners in the earlier phases of their professional trajectory, domain schemata remain fluid and underdeveloped. In these cases, deliberate prompt structuring appears to serve as a supportive external proxy, being closely associated with lower perceived mental effort before the high-stakes output validation stage. As expertise deepens into the intermediate range, practitioners are actively consolidating schemata while simultaneously navigating the probabilistic unpredictability inherent in LLM output. For them, structured prompts continue to correspond with lower verification overhead, acting as an external support for cognitive tasks that their partly-formed schemata cannot yet absorb autonomously.

Among seasoned experts, however, the direct association between prompt strategy and cognitive offloading appears numerically less prominent. Rather than indicating a failure of strategic interaction, this divergence highlights a unique skill-leveling effect and potential domain schema interference triggered by the algorithmic black box. Veterans possess richly interconnected concept networks; however, when confronted with the probabilistic and opaque nature of LLMs, their traditional expertise may trigger cognitive resistance or algorithmic aversion. Consequently, what experts require is not merely basic procedural certainty, but rather advanced logical verification tools. In this context, prompt strategy preference is primarily associated with higher AI interaction self-efficacy—reflecting a sense of strategic agency over an opaque generative system. For these experts, it is this elevated self-efficacy that is most strongly linked to lower cognitive load. However, as the multi-group structural equivalence tests robustly demonstrated, this exploratory trend of “attenuated direct cognitive offloading and relatively increased self-efficacy mediation” has not reached the threshold for statistical divergence. Therefore, it cannot be formalized as a profound “stage-specific functional evolution” driven by accumulated experience.

Taken together, these patterns robustly confirm that the overarching mechanism for managing LLM-related cognitive demands is fundamentally stable and universal across experience levels. Traditional domain expertise does not impose a significant boundary moderation effect on these foundational psychological pathways. The core structural dependencies remain highly intact—a finding with meaningful implications for how organizations should uniformly calibrate training priorities and interface support across the career span (Vasconcelos et al., 2023).

### 5.4 Theoretical contributions and practical management implications

From the intersection of information system use and applied psychology, this study quantitatively expands the functional attributes of prompt strategy configuration at the human cognitive level. Previous computer science literature mostly viewed prompt engineering as a data optimization method to improve machine generation accuracy; this empirical study shows that this behavior also constitutes an important regulatory mechanism for human users when processing high-intensity knowledge collaboration tasks. This finding provides theoretical and data references for introducing cognitive ergonomics into new operational paradigms of digital tools (Yao et al., 2026). By unpacking the psychological dimensions of this paradigm, the theoretical contributions of this study crystallize into three specific propositions: (1) It formally extends the applicability boundary of Cognitive Offloading Theory into the uncertain knowledge collaboration of generative AI; (2) It provides a contextualized revision of the “expert advantage” assumption in traditional Cognitive Load Theory (CLT) by highlighting the potential schema interference and skill-leveling effect of AI; and (3) It offers robust, large-sample empirical evidence identifying AI interaction self-efficacy as a crucial “psychological buffer” within human-computer interaction (HCI).

When translated into practical scenarios of corporate management and language service organizations, this conclusion provides suggestions for the design of employee ergonomic support systems in the era of generative AI. Currently, many training structures are limited to teaching how to write instructions to pursue higher text quality, while ignoring the risk of practitioner cognitive burnout hidden in model operations. At the organizational intervention level, the establishment of AI interaction self-efficacy should be included in the core objectives of training, consciously guiding employees to distribute working memory pressure through upfront strategic methods. For system tool developers, structured instruction templates (such as terminology constraint plug-ins or workflow splitting wizards) specific to the domain can be embedded in the interactive interface design. By directly lowering the threshold for constructing high-level prompts, this can enhance the operator's efficacy beliefs during the empowerment phase, thereby systematically mitigating task fatigue among professional workers in intelligent collaboration (Srinivasaprasad et al., 2025).

### 5.5 Limitations and future research directions

Limited by the research design and data collection methods, this empirical work has certain limitations. First, this study adopts a cross-sectional design. Although the structural equation model is constructed based on a theory-driven logic chain of “prompt strategy → self-efficacy → cognitive load” and the empirical data show high structural fit, there are still boundary constraints on rigorous methodological causal inference. Especially in real human-computer interaction, the possibility of two-way causality or feedback loops cannot be completely ruled out: practitioners who exhibit “high load” or are in a state of “cognitive depletion” may actively give up writing complex chain-of-thought instructions due to the exhaustion of psychological resources, thereby reversely leading to the weakening of “prompt strategy preference” (i.e., the reverse paths of N → P or N → SE). This mutually causal structural relationship mapping needs to be clarified by more rigorous causal separation techniques.

Second, regarding measurement tools, the limitation of the Raw NASA-TLX retrospective cumulative measurement adopted in this study is that it flattens the dynamic fluctuations of load over time. This one-dimensional summarization cannot open the “black box” of the interaction process, making it difficult to accurately depict the instantaneous psychological stress peaks of individuals when facing hallucinated outputs. In addition, the reliance of the entire scale on self-reported data, even if it passes the common method bias test, may still have memory bias effects during subjective cognitive recall.

Future research can supplement the observation methodology with tracking intervention tests characterized by longitudinal interventions. By assigning strategy training tasks to different subject groups and comparing the trend of indicators before and after the intervention, the strength of causal inference can be enhanced. In terms of measurement environments, future studies can attempt to break through the single subjective questionnaire model, collect users' objective interaction logs (such as keystroke logging and interaction pause times) in actual human-computer collaborative desktop tasks, and even introduce neuroergonomic physical indicators such as eye-tracking or electroencephalography (EEG) to achieve cross-modal triangulation of self-reported parameters and objective physiological indicators, thereby advancing the in-depth exploration of cognitive depletion mechanisms in human-computer collaboration in this field.

## 6 Conclusion

The deep intervention of generative artificial intelligence in knowledge workflows has substantially reshaped the execution processes of professional groups such as terminology workers, shifting part of the pressure of quality control and logical correction to the human-computer interaction interface. By constructing a multivariate verification model combined with latent profile extraction, this study confirms that when facing the uncertainty challenges brought by large language model outputs, practitioners' operational preferences in prompt strategies are not only external interventions on the precision of text output but also participate more deeply in the internal regulation process of users' subjective cognitive load. Empirical data show that groups tending to use high-level strategy combinations exhibit more significant cognitive offloading effects. In this transmission link, AI interaction self-efficacy, as a belief in system control in digital contexts, plays a stable and key mediating role; meanwhile, this regulatory path is not constrained by the users' professional seniority in traditional professional areas. Crucially, while this “strategy-efficacy-load” mechanism was validated within terminology work, its underlying logic extends naturally to broader knowledge-intensive industries characterized by strict semantic demands and high error costs—such as legal contract drafting, medical diagnostic assistance, and financial compliance review. The integrated findings suggest that developing appropriate prompt regulation skills and accumulating system interaction self-efficacy in this process constitute an effective, universal framework to cope with mental load challenges and seek a rebalancing of cognitive resources under the current background of intelligent technology transition.

## Data Availability

The original contributions presented in the study are included in the article/Supplementary material, further inquiries can be directed to the corresponding author.
