# Supplementary material for: Generative artificial intelligence and terminology work: structural associations of prompt strategy preference, cognitive load, and self-efficacy
Source: Front Psychol. 2026 Oct 30;17:1929666. doi: 10.3389/fpsyg.2026.1929666 (PMC13759274; doi:10.3389/fpsyg.2026.1929666)
Supplement: Supplementary file 1 [file Data_Sheet_1.docx]

**Supplementary Material S2: Extended Statistical Analysis Syntax and Sensitivity Results**

To ensure methodological transparency in accordance with Open Science practices, this document provides the core analysis syntax used in this study, alongside the detailed outputs of supplementary analyses (Pilot EFA, HTMT, ULMC, and Measurement Invariance) requested during the review process.

The underlying analytical dataset is strictly mapped and provided as Dataset_S2_Anonymized.dat. Note: In this publicly shared dataset, demographic variables (such as age and education) have been independently shuffled to strictly protect participant privacy. Consequently, executing the following syntax with the public dataset will yield approximate structural trajectories rather than exact replications of the covariate-adjusted SEM estimates reported in the manuscript.

#### **1. SPSS Syntax for Data Computation and Reliability**

This section details the specific reverse-scoring logic and the generation of composite covariates (including Task Complexity and System Trust) mapping onto the structural model adjustments.

* 1.1 Reverse Scoring for NASA-TLX item (N6) and Trust items (TR3, TR4).
COMPUTE N6_rev = 8 - N6.
COMPUTE TR3_rev = 8 - TR3.
COMPUTE TR4_rev = 8 - TR4.
EXECUTE.

* 1.2 Unweighted Arithmetic Aggregation for Latent / Composite Variables.
COMPUTE PESP = MEAN(P1, P2, P3, P4, P5, P6, P7).
COMPUTE TLX_Overall = MEAN(N1, N2, N3, N4, N5, N6_rev).
COMPUTE SelfEfficacy = MEAN(SE1, SE2, SE3, SE4, SE5).
COMPUTE TaskComp_mean = MEAN(T1, T2, T3, T4).
COMPUTE TermComp_mean = MEAN(C1, C2, C3, C4, C5).
COMPUTE SysTrust_mean = MEAN(TR1, TR2, TR3_rev, TR4_rev, TR5).
EXECUTE.

* 1.3 Reliability Analysis for Prompt Strategy Preference (PESP).
RELIABILITY
 /VARIABLES=P1 P2 P3 P4 P5 P6 P7
 /SCALE('PESP-T') ALL
 /MODEL=ALPHA
 /STATISTICS=DESCRIPTIVE SCALE CORR.

#### **2. Pilot Study: Exploratory Factor Analysis (EFA) for PESP-T**

Prior to the formal study, a pilot test (*N* = 120) was conducted to evaluate the unidimensionality of the newly developed Prompt Engineering Strategy Preference Scale (PESP-T).

**KMO and Bartlett’s Test:** KMO = 0.882, Bartlett‘s χ² = 385.45(*p* < .001).

**Factor Extraction (Principal Component Analysis):** A single dominant factor emerged (Eigenvalue = 4.57), explaining 65.4% of the total variance. All 7 items exhibited strong factor loadings ranging from 0.71 to 0.86, confirming the structural validity of PESP-T as a unidimensional construct.

#### **3. Heterotrait-Monotrait Ratio (HTMT) Assessment**

To rigorously evaluate discriminant validity, the HTMT matrix was generated. All values are strictly below the conservative threshold of 0.85, establishing strong discriminant characteristics across all theoretical constructs.

| Latent Variables | PESP | Self-Efficacy | TLX Load | Task Complexity | Trust | TermComp | OutputQual |
| --- | --- | --- | --- | --- | --- | --- | --- |
| PESP | - |  |  |  |  |  |  |
| Self-Efficacy | 0.312 | - |  |  |  |  |  |
| TLX Load | 0.178 | 0.285 | - |  |  |  |  |
| Task Complexity | 0.089 | 0.045 | 0.291 | - |  |  |  |
| Trust | 0.185 | 0.062 | 0.081 | 0.095 | - |  |  |
| TermComp | 0.191 | 0.277 | 0.099 | 0.022 | 0.008 | - |  |
| OutputQual | 0.375 | 0.428 | 0.302 | 0.066 | 0.024 | 0.156 | - |

#### **4. Unmeasured Latent Method Construct (ULMC) Analysis**

To rigorously test for Common Method Bias (CMB) beyond Harman’s single-factor test, an unmeasured latent method factor was introduced into the CFA model.

**Baseline CFA Fit:** *χ*² = 972.8, *df* = 608, *χ*²/*df* = 1.60, CFI = 0.985, RMSEA = 0.018

**ULMC Model Fit:** *χ*² = 959.0, *df* = 607, *χ*^2^ /*df* = 1.58, CFI = 0.988, RMSEA = 0.016

**Conclusion:** The inclusion of the method factor yielded negligible improvements(ΔCFI = 0.003 < 0.01, ΔRMSEA = 0.002 < 0.015). Substantive trait factor loadings remained robust (Δλ < 0.05), indicating CMB is not a threat to the structural integrity.

#### **5. NASA-TLX Sensitivity Analysis (Excluding N5 and N6)**

To address potential theoretical concerns regarding whether physical demand (N5) and performance (N6) dimensions might distort mental load evaluations, the structural model was re-estimated using a restricted 4-item cognitive load construct (N1, N2, N3, N4).

The direct path (PESP → Load) remained significant: *β* = −0.115, 95% CI [−0.176, −0.054], *p* = .008.

The indirect path (PESP → Self-Efficacy → Load) remained significant: $\mathrm{Indirect}$ *β* = −0.075$,$ *95% CI [−0.106, −0.044],* *p* < .001$\text{. }$
This sensitivity model confirms that the overall structural associations remain highly robust regardless of the inclusion of ergonomic and performance dimensions.

#### **6. AMOS Syntax for Adjusted Structural Equation Model (SEM)**

The fully adjusted SEM appropriately retained all demographic and contextual covariates (Age, Education, Task Complexity, and System Trust) mapped onto the endogenous variables exactly as reported in the manuscript.

! Measurement Model paths
PESP =~ P1 + P2 + P3 + P4 + P5 + P6 + P7
SelfEff =~ SE1 + SE2 + SE3 + SE4 + SE5
TLX =~ N1 + N2 + N3 + N4 + N5 + N6_rev

! Structural Model (Direct and Indirect Hypotheses)
SelfEff <--- PESP
TLX <--- PESP
TLX <--- SelfEff

! Fully Adjusted Covariates Evaluated (All retained)
TLX <--- Age
TLX <--- Edu
TLX <--- TaskComp_mean
TLX <--- SysTrust_mean
SelfEff <--- Age
SelfEff <--- Edu

! Bootstrapping setup for Mediation
Bootstrap Samples = 5000
Confidence Interval = 95% (Bias-corrected)

#### **7. Mplus Syntax for Latent Profile Analysis (LPA)**

*Note: The NAMES ARE variable list strictly matches the 44 sequential columns of the uploaded Dataset_S2_Anonymized.dat file (formatted without headers to facilitate immediate Mplus execution).*

TITLE: Latent Profile Analysis for Prompt Strategy Preference (3-Profile Model)
DATA:
 FILE IS Dataset_S2_Anonymized.dat;
VARIABLE:
 ! Ensuring perfect match with 44 columns of the .dat file
 NAMES ARE
 ID Q1_Gend Q2_Age Q3_Edu Q4_Job Q5_Exp Q6_Freq
 C1 C2 C3 C4 C5 T1 T2 T3 T4
 TR1 TR2 TR3 TR4 TR5
 P1 P2 P3 P4 P5 P6 P7
 N1 N2 N3 N4 N5 N6
 SE1 SE2 SE3 SE4 SE5
 Q1 Q2 Q3 Q4 Q5;

 USEVARIABLES ARE P1 P2 P3 P4 P5 P6 P7;
 CLASSES = c(3);
ANALYSIS:
 TYPE = MIXTURE;
 STARTS = 500 100;
 STITERATIONS = 50;
MODEL:
 %OVERALL%
OUTPUT:
 TECH11; ! Lo-Mendell-Rubin LRT
 TECH14; ! Bootstrapped LRT
 ENTROPY;

***8. Measurement Invariance Analysis Results Across Experience Groups***
To confirm that the measurement model operates equivalently across user cohorts with different professional experience levels (novices, intermediates, and experts), consecutive measurement invariance constraints were tested. The results below demonstrate that changes in fit indices are well within the acceptable thresholds (ΔCFI ≤ -0.01 and ΔRMSEA ≥ 0.015), supporting the robustness of subsequent multigroup structural comparisons.

| Invariance Model | *χ*² | *df* | CFI | RMSEA | SRMR | Model Comparison | ΔCFI | ΔRMSEA |
| --- | --- | --- | --- | --- | --- | --- | --- | --- |
| M1: Configural (No constraints) | 1856.4 | 1824 | 0.985 | 0.018 | 0.038 | - | - | - |
| M2: Metric (Loadings constrained) | 1898.3 | 1888 | 0.984 | 0.018 | 0.040 | M2 vs M1 | -0.001 | 0.000 |
| M3: Scalar (Intercepts constrained) | 1952.7 | 1952 | 0.982 | 0.019 | 0.042 | M3 vs M |  |  |
